# Epibrassinolide and melatonin co-treatment enhances salt tolerance in tomato plants by coordinating photosynthetic efficiency, proline accumulation, and antioxidant defense

**DOI:** 10.3389/fpls.2025.1621310

**Published:** 2025-09-17

**Authors:** Mohammad Yusuf, Tanveer Alam Khan, Taiba Saeed

**Affiliations:** ^1^ Biology Department, College of Science, United Arab Emirates University, Al Ain, United Arab Emirates; ^2^ Department of Biosciences, Integral University, Lucknow, Uttar Pradesh, India

**Keywords:** brassinosteroids, melatonin, salinity, RuBisCO, photosynthesis, electrolyte leakage

## Abstract

Melatonin (ML) and 24-epibrassinolide (EBL) are both known to help plants cope with abiotic stress. However, their interactive effects on the physiological performance of plants under high salinity have not been fully explored. To address this gap, the present study was conducted to investigate the combined role of ML and EBL in enhancing salt stress tolerance in tomato plants. The effects of ML and EBL, applied individually and in combination, were assessed under high salinity conditions by measuring plant growth, photosynthetic efficiency, stress-related biomarkers, proline metabolism, and antioxidant enzyme activity. The results showed that applying ML before stress and EBL after stress significantly reduced the accumulation of ROS. This effect was achieved by boosting antioxidant activity and regulating key enzymes involved in proline metabolism. These biochemical adjustments, such as enhanced antioxidants and altered proline metabolism, led to reduced lipid peroxidation, lower hydrogen peroxide (H_2_O_2_) levels, and decreased electrolyte leakage. Furthermore, the interactive treatment improved photosynthetic efficiency and growth by enhancing the activity of RuBisCO, a key enzyme in carbon fixation. Overall, this study provides novel insights into the synergistic role of EBL and ML in conferring salt stress tolerance through improved proline metabolism, redox regulation, and photosynthetic performance. The findings suggest that the co-application of ML and EBL holds significant potential as an eco-friendly strategy to enhance salinity tolerance in tomato plants, thereby contributing to sustainable agricultural practices under saline conditions.

## 1 Introduction

By 2050, the global population is expected to exceed 9.6 billion, necessitating a 70% increase in food production (FAO, 2017). This demand poses a significant challenge for agriculture, especially in regions already constrained by environmental stressors. Soil salinity has emerged as a critical abiotic stress, particularly in arid and semi-arid regions, where it severely hampers crop productivity and land sustainability (Deolu-Ajayi et al., 2024; Hassan et al., 2025). Salinization affects over 20% of irrigated land globally and continues to expand, primarily due to unsustainable irrigation and poor drainage practices (Selma, 2025). Each year, hundreds of thousands of hectares become agriculturally unproductive due to salt accumulation (FAO, 2017). Salinity imposes osmotic and ionic stress, disrupting water uptake and leading to toxic levels of sodium (Na^+^) and chloride (Cl^-^), nutrient imbalance, and cellular injury. Plants respond to this stress in two stages: an early osmotic phase causing reduced cell expansion and stomatal closure, followed by a chronic ionic phase that leads to ion toxicity, senescence, and metabolic suppression (Selma, 2025). Photosynthesis, which contributes up to 90% of plant biomass, is highly sensitive to salinity. Reduced stomatal conductance limits CO_2_ assimilation, while excessive ROS production damages chloroplasts and enzymes (Tomaškinová et al., 2025). To counteract these effects, plants activate antioxidant systems and osmolyte synthesis pathways, including proline metabolism, to restore redox balance and maintain physiological functions (Nurhidayati et al., 2024). Recent studies emphasize the importance of integrated strategies such as bio-stimulants, salt-tolerant genotypes, and molecular breeding to enhance crop resilience under saline conditions (Hassan et al., 2025). These insights are vital for developing sustainable agricultural practices in the face of escalating soil salinity and global food insecurity.

Brassinosteroids (BRs), a group of steroidal plant hormones and essential regulators of key physiological activities, including cell division and elongation, vascular tissue differentiation, and floral development (Vriet et al., 2012). The discovery and characterization of BR-deficient and BR-insensitive mutants in recent years have significantly advanced our understanding of BR signaling pathways and their contribution to plant growth and the development of improved agronomic traits (Nolan et al., 2020). BRs are known to promote both cell division and expansion, and they function by interacting with other hormone signaling networks at the transcriptional level to coordinate plant growth responses (Gudesblat and Russinova, 2011; Khan et al., 2023). In metabolically active tissues, BRs exhibit concentration and sensitivity-dependent effects, regulating growth and development while modulating antioxidant defenses, especially under stress-induced overproduction of ROS. BRs have also been reported to enhance plant tolerance to several stresses, including salinity, drought, extreme temperatures, and heavy metal toxicity (Bajguz and Hayat, 2009; Khan et al., 2024). Despite these advances, the interaction between BRs and other phytohormones under high salinity conditions, particularly about their combined effects on proline metabolism and antioxidant systems, remains poorly understood. On the other hand, melatonin (ML), a recently identified plant bio-regulator, has attracted considerable attention due to its diverse physiological roles, such as delaying leaf senescence, promoting root and shoot development, enhancing nutrient uptake, and improving heat stress tolerance (Ahammed et al., 2018). The role of ML in plant stress physiology has been investigated using both synthetic ML applications and transgenic approaches that elevate endogenous ML levels (Erland et al., 2018). Exogenous ML treatment has been demonstrated to protect plants from diverse abiotic stresses by mitigating damage caused by ROS (Karumannil et al., 2023; Yusuf et al., 2024). Specifically, ML-mediated stress tolerance has been linked to enhanced synthesis of antioxidants, activation of stress-responsive enzymes, regulation of polyamine metabolism, and efficient ROS scavenging (Ni et al., 2018). However, the potential crosstalk between BRs and ML in mediating plant tolerance to high salinity, particularly through the modulation of proline metabolism and antioxidant systems, has not yet been elucidated. Exploring this interaction may uncover novel strategies for enhancing stress resilience in crops under saline environments. Proline serves as a key osmoprotectant and antioxidant, playing a central role in mitigating oxidative damage by scavenging ROS, thereby enhancing plant resilience to abiotic stresses and reducing associated agricultural losses. Despite its importance, the regulatory mechanisms underlying proline metabolism in response to external stimuli remain incompletely understood.

This study aims to investigate the modulation of proline metabolism under high salinity stress following exogenous co-application of ML and/or EBL. Specifically, the research evaluates the capacity of ML to enhance salt stress tolerance in the presence of EBL, with a particular focus on photosynthetic performance, proline accumulation, and antioxidant defense responses. 

## 2 Materials and methods

### 2.1 Plant material

Seeds of *Lycopersicum esculentum* (tomato) were obtained from the local seed market at Al Ain Central Market, Al Ain, UAE. Uniform, healthy seeds were selected and surface-sterilized using a 1% sodium hypochlorite solution for 10 minutes, followed by thorough rinsing with deionized water.

### 2.2 Hormone preparation

ML and EBL were procured from Sigma-Aldrich, USA. Stock solutions of ML (1 mM) and EBL (10^-4^ M) were prepared separately by dissolving the required amounts of each compound in 5 mL of ethanol, followed by dilution to 100 mL with deionized water in volumetric flasks. Working concentrations of ML (100 µM) and EBL (0.01 µM) were obtained by appropriate dilution of the respective stock solutions. Tween-20 was added as a surfactant before application.

### 2.3 Source of salt stress

Sodium chloride (NaCl) was used to induce salt stress. A 1 M stock solution was prepared by dissolving the required amount of NaCl in 10 mL of deionized water in a 100 mL volumetric flask, and the volume was adjusted to the mark with deionized water. The desired working concentration of 300 mM NaCl was obtained by diluting the stock solution accordingly.

### 2.4 Experimental design and treatment patterns

Tomato seeds were surface-sterilized and sown in pots filled with a standardized potting mix to establish nursery seedlings. After 20 days, healthy seedlings were carefully transplanted into 40 plastic pots containing the same potting medium. These pots were arranged in a completely randomized design (CRD) within a climate-controlled greenhouse. The experimental setup consisted of eight treatment groups, each containing five replicate pots with three plants per pot. All measurements were performed in triplicate for each treatment.

The treatment groups were organized as follows:

Set I served as the untreated control (no EBL, ML, or NaCl).
Set II involved foliar application of 0.01 μM EBL for five days, initiated 20 days after transplanting (post-NaCl exposure).
Set III received a foliar application of 100 μM ML for five days, beginning five days after transplanting (pre-NaCl exposure).
Set IV involved a combination of EBL and ML treatments.
Set V was initiated at 10 days post transplanting by adding 300 mM of NaCl through soil for five consecutive days.
Set VI combined salt stress with EBL treatment (Set II + Set V).
Set VII combined salt stress with ML treatment (Set III + Set V).
Set VIII integrated salt stress with both EBL and ML treatments (Set IV + Set V).

Each foliar treatment was administered through a sprayer delivering approximately 1 ml per spray, applied three times to each plant. All plants were maintained until 40 days after transplanting, and samples were collected at 60 days post-sowing for analysis of growth traits, photosynthetic efficiency, oxidative stress indicators, and the activities of enzymes involved in proline metabolism and antioxidant defense.

### 2.5 Growth characteristics

Plant samples from each treatment were carefully uprooted along with the surrounding soil and gently washed in a beaker filled with tap water to remove adhering soil particles. The cleaned samples were blotted dry using paper towels. Shoot and root lengths were measured using a meter scale. The same plants were then placed in an oven at 70°C for 96 hours for drying, after which their dry weights were recorded.

The leaf area of each leaf from each treatment was determined by tracing on a graph sheet, and the data were recorded. The leaf area per plant was computed by calculating the average leaf area of the plant.

Leaf relative water content (RWC) was measured using fresh leaf discs (2 cm in diameter), excluding the midrib. The discs were immediately weighed to record their fresh weight and then floated on deionized water in Petri dishes for 24 hours in the dark to achieve full turgidity. After saturation, excess surface water was gently blotted off, and the turgid weight was recorded. The discs were then oven-dried at 60°C for 72 hours to obtain the dry weight. RWC was calculated using the following formula:


LRWC = (FM−DM)/(TM−DM) × 100

Where, FM = Fresh mass; DM = Dry mass; TM= Turgor Mass

### 2.6 Chlorophyll content and photosynthetic traits

Chlorophyll content in the leaves was measured under natural conditions using a SPAD chlorophyll meter (SPAD-502; Konica Minolta Sensing, Inc., Japan).

All photosynthetic parameters, including net photosynthetic rate, stomatal conductance, and internal CO_2_ concentration, were measured following the methodology described in our previous study (Yusuf et al., 2016).

### 2.7 Stress biomarkers

Lipid peroxidation, electrolyte leakage, and hydrogen peroxide (H_2_O_2_) content in all plant samples were analyzed using the methods described by Yusuf et al. (2011).

### 2.8 Biochemical attributes

Soluble sugar content in dried leaf samples was assessed using 80% ethanol extraction, following the method of Krishnaveni et al. (1984) with slight modifications.

The activities of antioxidant enzymes catalase (CAT), peroxidase (POX), and superoxide dismutase (SOD) were measured as described in our previous study (Naz et al., 2015). Glutathione reductase (GR) activity was measured as mentioned in Yusuf et al. (2011).

Proline accumulation was also determined according to the procedure outlined in Naz et al. (2015).

Activities of key enzymes involved in proline metabolism, including Rubisco, pyrroline-5-carboxylate synthase (P5CS), delta-ornithine aminotransferase (δ-OAT), and proline dehydrogenase (ProDH), were analyzed following the method reported in our earlier study (Yusuf et al., 2017).

### 2.9 Statistical analysis

The collected data were statistically analyzed using the Statistical Package for the Social Sciences (SPSS, version 17.0 for Windows; Chicago, IL, USA). Standard error was calculated, and analysis of variance (ANOVA) was conducted to assess differences among treatment means. The least significant difference (LSD) test was applied at a significance level of *P ≤ 0.05* to determine statistically meaningful differences.

## 3 Results

### 3.1 Growth performance

**Figure 1:**
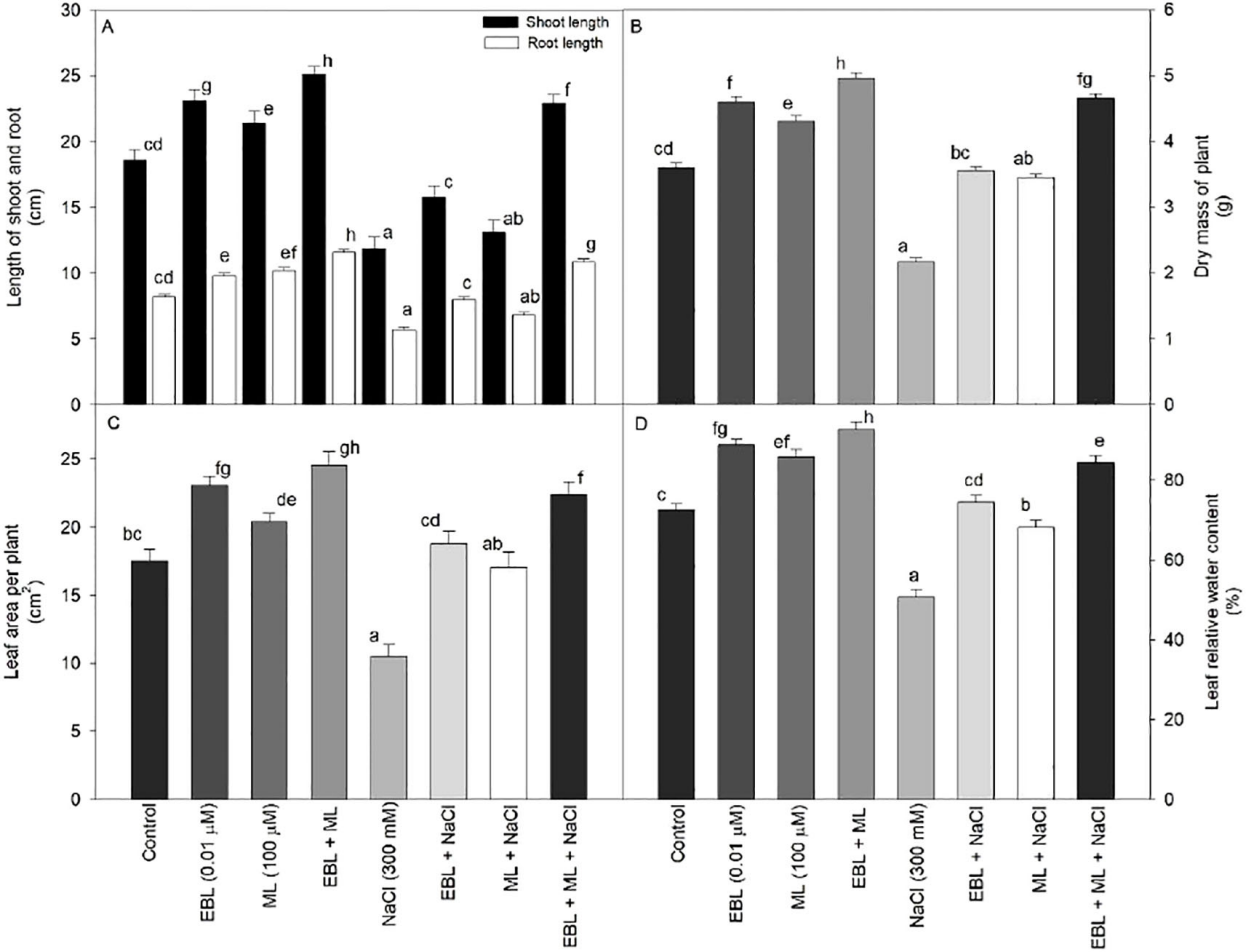
Effect of 24-epibrassinolide (EBL; 0.01 µM) and/or melatonin (ML; 100 µM) induced changes in **(A)** shoot and root length, **(B)** dry mass of plant, **(C)** leaf area per plant, and **(D)** leaf relative water content under excess level of salt (NaCl; 300 mM) in tomato plants at 60 days after sowing. All the data are the mean of five replicates (n=5) and vertical bars show standard errors (± SE). Different letters indicate a significant difference between control and treatment by LSD test *p* ≤ 0.05.

Growth performance was assessed through measurements of shoot and root lengths, plant dry mass, and leaf area. As shown in **Figures 1A–D**, exposure to high salinity stress (300 mM NaCl) resulted in a significant decline across all growth indicators compared to the untreated control. However, treatments with EBL and ML applied individually or in combination significantly alleviated the inhibitory effects of salinity, promoting shoot and root elongation, biomass accumulation, and leaf area expansion. Notably, the co-application of EBL and ML produced the most substantial improvements, increasing shoot length by 34.94%, root length by 41.46%, plant dry mass by 37.77%, and leaf area by 40.0% in comparison to their control plants. While single applications of either EBL or ML led to moderate recovery, their combined use was more effective in counteracting the detrimental impact of 300 mM NaCl, bringing growth parameters close to those observed under non-stress conditions.

### 3.2 Physiological performance

**Figure 2:**
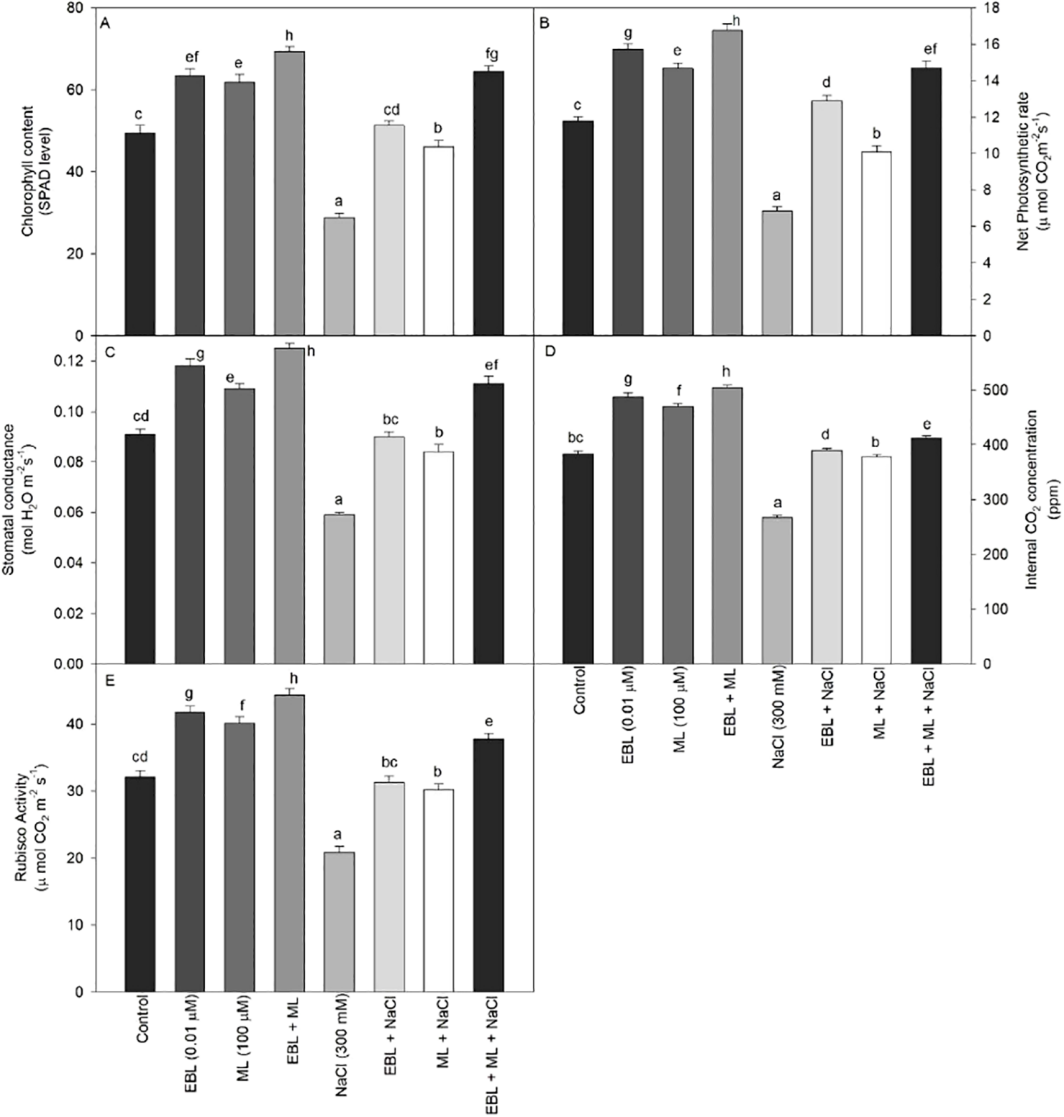
Effect of 24-epibrassinolide (EBL; 0.01 µM) and/or melatonin (ML; 100 µM) induced changes in **(A)** chlorophyll content (SPAD level), **(B)** net photosynthetic rate, **(C)** stomatal conductance, **(D)** internal CO_2_ concentration, and **(E)** Rubisco activity under excess level of salt (NaCl; 300 mM) in tomato plants at 60 days after sowing. All the data are the mean of five replicates (n=5) and vertical bars shows standard errors (± SE). Different letters indicate a significant difference between control and treatment by LSD test *p ≤ 0.05*.

All measured photosynthetic parameters, including net photosynthetic rate, stomatal conductance, internal CO_2_ concentration, SPAD chlorophyll content, and Rubisco activity, were significantly suppressed under salt stress induced by 300 mM NaCl (**Figures 2A–E**). Specifically, NaCl treatment led to a 35.0% reduction in Rubisco activity and a 42.37% decrease in net photosynthetic rate compared to the control plants. In contrast, the application of EBL and/or ML markedly improved these parameters under salinity stress. While individual treatments with EBL or ML partially alleviated the negative effects of salt stress, the combined application of both significantly restored and even enhanced photosynthetic performance. Notably, stressed plants treated with both EBL and ML exhibited full recovery in terms of net photosynthetic rate, chlorophyll content, and Rubisco activity, with values surpassing those observed in control plants. These findings highlight the synergistic role of EBL and ML in protecting and enhancing photosynthetic machinery under saline conditions.

### 3.3 Stress biomarkers

**Figure 3:**
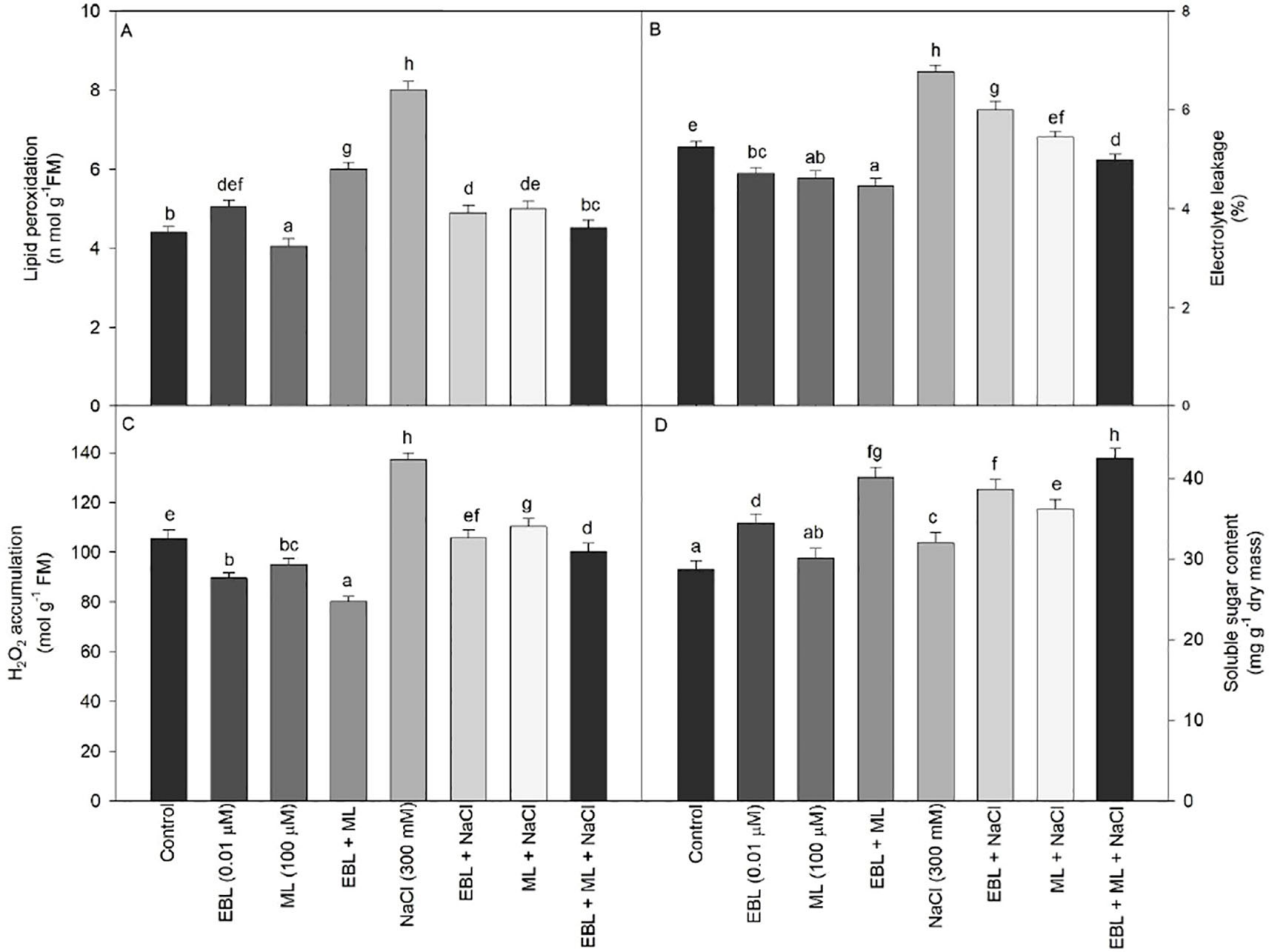
Effect of 24-epibrassinolide (EBL; 0.01 µM) and/or melatonin (ML; 100 µM) induced changes in **(A)** lipid peroxidation, **(B)** electrolyte leakage, **(C)** H_2_O_2_ content, and **(D)** soluble sugar content under excess level of salt (NaCl; 300 mM) in tomato plants at 60 days after sowing. All the data are the mean of five replicates (n=5) and vertical bars shows standard errors (± SE). Different letters indicate a significant 70 difference between control and treatment by LSD test *p ≤ 0.05*.

Various parameters, including lipid peroxidation, electrolyte leakage, and H_2_O_2_ content, were evaluated to assess the stress induced by NaCl. All these parameters exhibited a significant increase at 300 mM NaCl (**Figures 3A–C**). However, the combined treatment of EBL and ML also elevated lipid peroxidation compared to the control plants. Conversely, under salt stress, the levels of electrolyte leakage, lipid peroxidation, and H_2_O_2_ content decreased when plants were treated with the EBL and ML combination.

The soluble sugar content consistently increased across all treatments compared to the control plants. Notably, exogenous application of EBL significantly enhanced soluble sugar levels, with the highest content observed in plants subjected to salt stress and treated with the EBL+ML combination, showing a 47.99% increase over control plants (**Figure 3D**).

### 3.4 Proline metabolism

**Figure 4:**
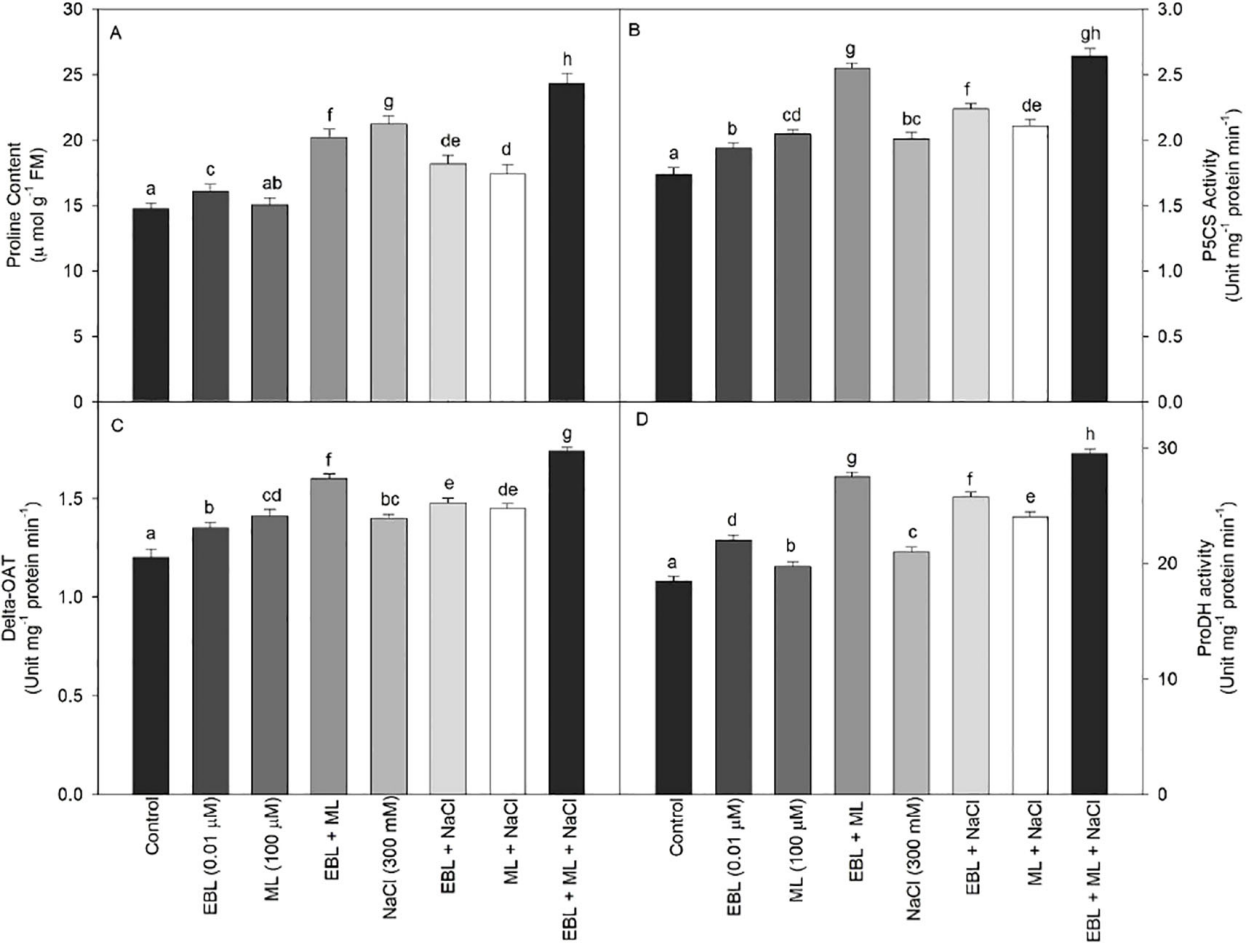
Effect of 24-epibrassinolide (EBL; 0.01 µM) and/or melatonin (ML; 100 µM) induced changes in **(A)** proline content, **(B)** P5CS activity, **(C)** δ-OAT activity, and **(D)** ProDH activity under excess level of salt (NaCl; 300 mM) in tomato plants at 60 days after sowing. All the data are the mean of five replicates (n=5) and vertical bars shows standard errors (± SE). Different letters indicate a significant difference between control and treatment by LSD test *p ≤ 0.05.*.

Proline metabolism involves the activities of P5CS, δ-OAT, and proline dehydrogenase, along with proline content. As shown in **Figures 4B–D**, the activities of all enzymes associated with proline metabolism increased across all treatments compared to the control plants. Treatment with EBL and ML individually also enhanced enzyme activities, but the combination of EBL and ML resulted in higher enzyme activity levels than either treatment alone. The highest enzyme activities were observed in plants grown under salt stress and treated with the EBL and ML combination.

Under salt stress, plants treated with EBL and ML showed a marked rise in proline accumulation (**Figure 4A**). Although both hormones elevated proline levels, EBL alone induced a greater increase than ML. The highest boost, a 64.9% increase, was observed in plants subjected to 300 mM NaCl and co-treated with EBL and ML.

### 3.5 Antioxidant system (activities of CAT, POX, SOD, and GR)

**Figure 5:**
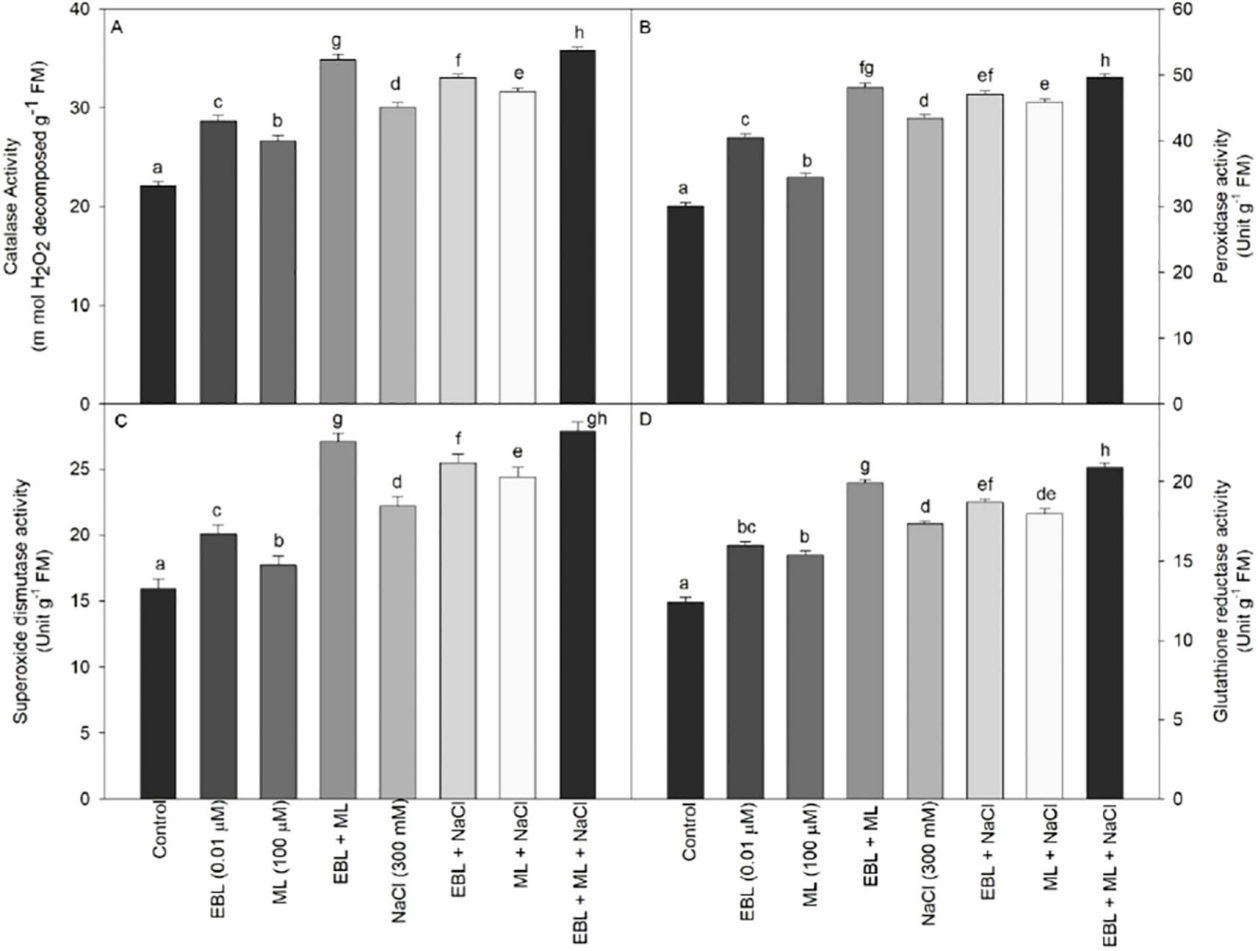
Effect of 24-epibrassinolide (EBL; 0.01 µM) and/or melatonin (ML; 100 µM) induced changes in **(A)** catalase, **(B)** peroxidase, **(C)** superoxide dismutase, and **(D)** glutathione reductase activities under excess level of salt (NaCl; 300 mM) in tomato plants at 60 days after sowing. Data are means + standard error of the five replicates (n = 5). Means that do not share a letter are significantly different at *P* ≤ 0.05 level according to Tukey's test.

Enzymatic assays shown in **Figures 5A–D** reveal that salt stress dramatically stimulates the activities of CAT, POX and SOD, with the lowest baseline activities observed in control plants. Application of EBL or ML alone significantly enhanced each enzyme’s activity, and their combined use produced an even greater, additive effect under saline conditions. Specifically, plants subjected to 300 mM NaCl and foliar treated with 0.01 µM EBL plus 100 µM ML exhibited the highest enzyme response, with CAT, POX, SOD, and GR activities increasing by 61.9%, 64.8%, 74.9%, and 67.8% respectively, compared to untreated controls.

## 4 Discussion

Plants frequently face environmental stresses such as drought, salinity, and temperature extremes, which elicit diverse responses at morphological, biochemical, and molecular levels. In the present study, exposure of tomato plants to 300 mM NaCl led to a significant increase in oxidative stress markers, including lipid peroxidation, electrolyte leakage, and H_2_O_2_ accumulation, indicators of severe cellular damage. These responses reflect impaired redox homeostasis and membrane destabilization under salinity stress. However, foliar application of ML, EBL, or their combination effectively mitigated these stress-induced elevations. This suppression of oxidative markers underscores their protective roles in enhancing cellular resilience. Notably, elevated levels of TBARS, a proxy for LPO, are a well-established hallmark of membrane deterioration under oxidative stress (Cavalcanti et al., 2007). Under salinity, increased ROS levels further compromise membrane integrity and may trigger programmed cell death through ionic imbalance and calcium-mediated signaling pathways (Demidchik et al., 2014). Salinity-induced disruption of electron transport chains in chloroplasts and mitochondria exacerbates ROS production, contributing to the accumulation of H_2_O_2_ in leaf tissues (Chawla et al., 2013). Our findings align with this mechanism, as salt-exposed plants exhibited heightened H_2_O_2_ levels (**Figure 3C**), which were significantly reduced by ML and EBL treatments. ML is known for its broad-spectrum antioxidative action across various stress contexts, including drought, cold, heat, and salinity, primarily through enhancement of the antioxidant defense system (Hasan et al., 2019; Kaya et al., 2020; Ahammed et al., 2020; Yusuf et al., 2024). In our study, ML application significantly lowered MDA, electrolyte leakage, and H_2_O_2_ levels in salt-stressed tomato, corroborating earlier findings in maize and other crops (Ahammed et al., 2020). Similarly, EBL application alleviated oxidative damage by reducing membrane injury markers, consistent with studies in wheat (Dong et al., 2017; Khan et al., 2024) and perennial ryegrass (Wu et al., 2017). BRs, including EBL, have been shown to enhance membrane stability through the upregulation of membrane-associated protective proteins (Li et al., 2012; Gao et al., 2015). These effects, as observed in our study, emphasize the role of BRs in stabilizing cellular structures and limiting ROS-mediated damage under saline conditions.

ROS are continuously generated in plant cells as byproducts of metabolic processes. Under optimal conditions, these are tightly regulated by a robust antioxidative system. However, under abiotic stresses such as high salinity, ROS production increases dramatically, overwhelming the cellular antioxidant machinery and leading to oxidative damage. This includes membrane lipid peroxidation, elevated H_2_O_2_ accumulation, and increased electrolyte leakage (**Figures 3A–C**), which impair cellular function and integrity. To combat oxidative stress, plants initiate a multifaceted defense response involving both enzymatic (CAT, POX, SOD, and GR) and non-enzymatic antioxidants. In our study, tomato plants subjected to 300 mM NaCl showed a pronounced increase in the activity of CAT, POX, SOD and GR. This response was significantly amplified by the application of ML, EBL, and most prominently by their combined treatment (**Figures 5A–D**). The upregulation of antioxidant enzymes under salinity is well-established. Early studies (Xue et al., 2008; Wu et al., 2011; Yusuf et al., 2024) demonstrated similar increases in enzymatic activity under NaCl stress. More recently, Sahu et al. (2025) reported that combined phytohormone treatments, including ML and BRs, enhance antioxidant capacity and confer improved salt tolerance in rice and wheat. Similarly, Li et al. (2025) found that exogenous ML application significantly boosted ROS-scavenging enzymes in salinity-stressed maize. ML is known to enhance antioxidative defense by upregulating genes encoding antioxidant enzymes through transcriptional activation, potentially mediated by ROS-responsive signaling pathways (Cui et al., 2017; Ahammed et al., 2020). Additionally, studies by Ali et al. (2024) highlight ML’s regulation of NAC and WRKY transcription factors under oxidative stress, further supporting its protective role. Moreover, EBL acts through a distinct but complementary mechanism and modulates stress-responsive transcription factors, including *BIN2* and *BES1/BZR1*, and activates downstream genes such as *DREB*, *WRKY*, *bZIP*, and *MYB/MYC* (Bartwal and Arora, 2020). This transcriptional network contributes to redox balance and improved stress tolerance. EBL has also been shown to influence the *det2* gene, enhancing the synthesis of protective enzymes under stress (Cao et al., 2005). Our data align with these findings, as EBL application significantly reduced markers of oxidative stress, including MDA, H_2_O_2_, and electrolyte leakage under salinity (**Figures 3A–C**). These observations affirm that strengthening antioxidant metabolism through ML and EBL applications is an effective strategy to mitigate salinity-induced oxidative damage. This approach holds promise for improving stress resilience in crop species, as evidenced by our results and corroborated by other recent studies (Fariduddin et al., 2014; Hasan et al., 2019; Sahu et al., 2025).

Proline serves a dual function in plant stress responses, acting as both an osmoprotectant and a redox buffer with significant antioxidant potential (Kavi Kishor and Sreenivasulu, 2014). Its metabolism is tightly regulated through key enzymes such as P5CS, P5CR, ProDH, and δ-OAT. During salt stress, the accumulation of proline helps maintain cellular osmotic balance and protects macromolecules from ROS-induced damage. Our findings show that salt-stressed tomato plants treated with a combination of ML and EBL exhibited significantly higher proline content and enhanced activity of P5CS, ProDH, and δ-OAT enzymes compared to untreated controls (**Figures 4A–D**). This suggests a coordinated upregulation of proline metabolism under the influence of ML and EBL. Similar results have been reported by Antoniou et al. (2017), who found that ML treatment increased both the transcription and activity of P5CS and P5CR in *Arabidopsis*. Vendruscolo et al. (2007) also demonstrated that P5CS overexpression enhances proline levels, reduces lipid peroxidation, and improves antioxidant defense. Likewise, Roosens et al. (2002) and Liu et al. (2014) reported that δ-OAT and P5CR play critical roles in stress-induced biomass enhancement and photosynthetic efficiency. EBL are known to stimulate proline accumulation under salinity by modulating the expression of biosynthetic genes (Özdemir et al., 2004; Zeng et al., 2010). Recently, Sahu et al. (2025) noted that exogenous EBL application in rice enhanced proline biosynthesis through transcriptional activation of stress-responsive pathways, while Xia et al. (2025) demonstrated that combined ML and BR treatment synergistically boosted proline metabolism and ROS scavenging capacity in maize.

**Figure 6:**
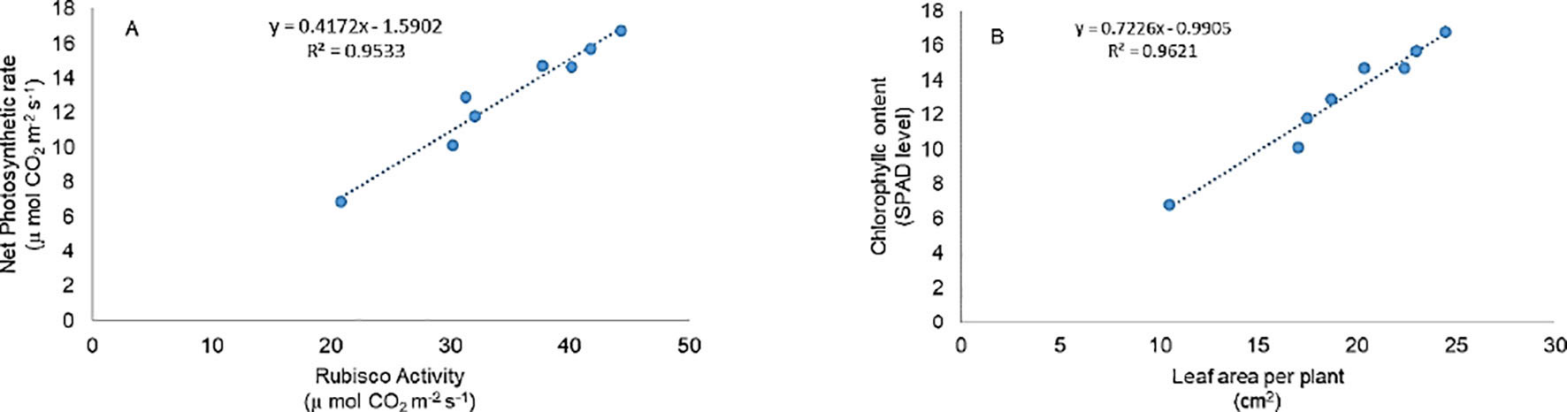
Pearson correlation coefficients were calculated to show the variable action of **(A)** net photosynthetic rate and Rubisco, and **(B)** leaf area per plant and chlorophyll content in tomato treatment. All the data are the mean of five replicates (n=5).

Photosynthesis is among the first cellular processes disrupted by salinity stress due to its sensitivity to redox imbalance (Biswal et al., 2011; Mathur et al., 2014). In our study, salinity severely impaired photosynthetic parameters, including chlorophyll content, net photosynthesis, stomatal conductance, and RuBisCO activity. However, application of ML and EBL, individually or in combination, significantly restored these traits (**Figures 2A–E**), alongside improving leaf relative water content (**Figure 1D**). The strong correlation observed between net photosynthetic rate and Rubisco activity (**Figure 6A**) further supports our finding that EBL and ML, either individually or in combination, significantly enhance photosynthetic efficiency. This recovery may stem from the stabilization of photosystem components and enhancement of Calvin cycle enzyme activity. Chloroplast dysfunction under salt stress, manifesting as thylakoid swelling and plastoglobuli accumulation, has been reported to impede light-harvesting efficiency (Meng et al., 2016). Salt-induced ABA signaling also limits water availability, reducing leaf relative water content and biomass (Davies et al., 2005; Zhang et al., 2006). ML has been shown to counter these effects by promoting RuBisCO activity, maintaining chlorophyll content, and enhancing the expression of photosynthesis-related genes (Zhao et al., 2015; Liang et al., 2015; Shi et al., 2015). In our data, ML-treated plants showed improved CO_2_ assimilation and sugar content even under stress (**Figures 2B–D**, **3D**), underscoring its regulatory influence on carbon metabolism. EBL also improved photosynthetic efficiency, likely through enhanced carboxylation capacity and upregulation of Calvin cycle enzymes, as previously reported by Yu et al. (2004) and confirmed in our results (**Figures 2A, C**). Zhao et al. (2017) similarly observed increased RuBisCO activity following EBL application under both stress and non-stress conditions. Furthermore, BRs have been linked to increased PSII efficiency and stabilization of chloroplast structure (Fariduddin et al., 2014; Nazir et al., 2021).

Salinity stress also triggered oxidative damage in our study, evidenced by increased lipid peroxidation, H_2_O_2_ accumulation, and electrolyte leakage (**Figures 3A–C**), ultimately reducing plant growth and productivity (Gupta and Huang, 2014). This was reflected in the reduced root and shoot lengths, lower biomass, and diminished leaf area (**Figures 1A–C**). A strong correlation between SPAD chlorophyll values and leaf area (**Figure 6B**) further supports the close link between photosynthetic efficiency and morphological development. The auxin-like activity of ML derived from the shared tryptophan precursor with IAA may underlie its role in nutrient uptake and growth promotion (Arnao and Hernández-Ruiz, 2007; Zhang et al., 2017). EBL, a potent growth regulator, operates through transcription factors like BZR1 and BES1/BZR2 to modulate growth-related genes even under stress (Gudesblat and Russinova, 2011). Our findings confirm that ML and EBL not only alleviate stress but also promote growth recovery, in line with previous reports (Fariduddin et al., 2014).

## 5 Conclusions

This study provides compelling evidence that ML and EBL, either alone or in combination, effectively mitigate salinity-induced oxidative stress in tomato plants. Their application enhances antioxidant capacity, activates proline metabolism, and reduces key stress markers such as lipid peroxidation, hydrogen peroxide levels, and electrolyte leakage. Importantly, the combined treatment of ML and EBL demonstrated superior effectiveness, preserving photosynthetic pigment content and enhancing RuBisCo activity under both stress and non-stress conditions. These findings suggest that the synergistic use of ML and EBL offers a promising, sustainable, and environmentally friendly strategy to boost salt tolerance and overall plant performance. This approach holds strong potential for practical application in saline agriculture, contributing to improved crop productivity and resilience in salt-affected regions.

As a future direction, it would be valuable to conduct transcriptomic or proteomic analyses to elucidate the precise molecular pathways and gene networks regulated by ML and BRs during salt stress. Additionally, time-course experiments across different developmental stages and field-level trials under varying salinity gradients could validate the efficacy and practical applicability of ML and EBL co-application in diverse tomato cultivars or other crops.

## Data Availability

The original contributions presented in the study are included in the article/supplementary material. Further inquiries can be directed to the corresponding author/s.
